# Dr. Cattell on Insanity

**Published:** 1848-07-01

**Authors:** Thomas Cattell

**Affiliations:** BRAUNSTON


### Original Communications, Translations, and Miscellaneous.

### ON INSANITY.

BY THOMAS CATTELL, M.D., M.R.C.S.E., BRAUNSTON.

### INSANITY DEMONSTRATED TO BE PURELY PHYSICAL IN ITS CHARACTER.

THE danger and importance of a disease is proportionate to the value of the function, or the rank which the particular organ or structure affected, holds in the animal economy. In the wide domain of medical science, there is no subject so profoundly or painfully interesting, or that demands so searching an investigation, as that which involves the normal manifestation of the mental attributes. Its phenomena have been inquired into, and every relation of the subject has received consideration and investigation commensurate only with their importance, in order that the treatment of a disturbed harmony of the attributes of mind may be made to rest on a scientific basis. With what results I shall not here stay to inquire,—suffice it to say, that though much has been done to prove its nature, and illustrate modes of treatment the best adapted to its melioration or cure, more yet remains to be accomplished.

If the cause of this be traced to its legitimate source, we shall find that it is to be referred to mistaken and absurdly erroneous views of the mind, and its relations to the brain. Thus—how can any one, regarding the mind as material, (the very *ex vi termini* of which involves a contradiction,) or as existing in the manner believed and inculcated by the advocates of phrenology, possess those accurate notions which a right understanding of the subject necessarily involves?

A proper knowledge of insanity must necessarily involve an accurate knowledge of the mind, apart from, and in relation to cerebral physiology and pathology; this, we fear, is not the case with many that we earnestly wish would examine the doctrines which they conceive to be true, and which they must have admitted on very slight foundation.

I have, however, no wish to expose to censure any proselyte of these contradictory and absurd doctrines. My object is the investigation and substantiation of the truth of the subject, without reservation on the one hand, or malediction on the other; and, if I refer to any opinion in connexion with the subject which would be likely to provoke to controversy, I shall not attach the name of its author.

To proceed: I affirm it to be a point essentially involved in this subject, and not admitting of doubt, that the mind is distinct in its nature and attributes from the structure and functions of the brain, and that no other entity but mind can manifest reason and consciousness.

This doctrine is directly antagonistic to that of a late writer on the subject, and who, from his position, must be well acquainted with every form in which the mental attributes are implicated by cerebral disease. Speaking of those distinctions to which we have just referred, he says,

"Both these opinions are extreme, and have their respective evils; if we acquiesce in the postulate, that nothing other than what is immaterial can display acts of intelligence, or possess consciousness, then it follows as a syllogistic necessity, that we must yield to animals the same spiritual entity which we assign to man, and thus, by distributing it generally, we apparently lessen its value, rob humanity of its distinguishing characteristic, and furnish the infidel with matter for triumph. Of these extreme opinions, the first leads to the fearful theory, that insanity is spiritual in its character, and is followed by a treatment cruel, inhuman, and atrocious; the latter overlooks the great power of psychical and moral influences, and is apt to give rise to the coarse, empirical, and dangerous practice of excessive bleedings, purgatives, vomitings, continued douches, and macerations."

Here is a great mistake,—it cannot be an extreme opinion to maintain that insanity is entirely physical in its character; it would not only be an extreme opinion to suppose the spiritual character of insanity, but the supposition will involve contradictions and absurdities without end. Besides, such a supposition confounds those radical and eternal distinctions which are absolutely essential to be made, and which the very name and nature of the subject presupposes distinct.

As to the various forms of insanity, these are no doubt referrible to the co-existence of certain modifying conditions of the brain, or system, other than that which primarily implicates the mental attributes.

We, therefore, unhesitatingly affirm, that intellectual insanity is purely physical in its character; or, in other words, that the particular implication of the mental attributes depends solely and altogether on a physical cause—as to the pathology of that cause, we leave for separate consideration.

To suppose that intellectual insanity can continue to manifest itself when the cerebral derangement on which it depends is cured; or, that this cerebral derangement can exist subsequent to the cure of the intellectual insanity, would be contradictory and absurd: as, in either case we must suppose that an effect survives the cause on which it depends for its own existence. But, since these suppositions are contradictory, and impossible because contradictory—since the certainty that the cure of intellectual insanity ensures the cure of the cerebral derangement, and the cure of this derangement ensures the cure of intellectual insanity, the effectuation of this in any instance must be the introduction of the patient into his original condition, *i. e.*, the healthy relation between his body and mind.

If the abnormal condition of the mental attributes continue subsequent to the cure of the cerebral derangement, it is evident that it could not have been produced by such derangement, because no effect can survive its cause.

As no other but a physical cause could produce intellectual insanity, the cure of this cause must necessarily extinguish those immediate and remote effects which ultimately depend upon it for their existence, and which can be supported in existence by no other cause. If either the immediate or remote effects of certain pathological states of the brain be supposed to continue in the case of those patients which have been cured, it might be asked on what cause or causes do these effects depend for their existence?—It is evident that every effect must have a cause, not only to produce but to continue it—a cause which is adequate to its production and continuance, and which must remain in union with the effect which it produced, and which still continues.

Unless we admit these general propositions, the term cause and effect become unintelligible and devoid of meaning.

In support of the position which has been taken, as to the purely physical character of intellectual insanity, we proceed to state that, as derangement of the brain is the cause of mental aberration, and the primary cause of all those effects which are included in, and result from it, whenever the derangement of brain is cured, we recognise the instant removal of the primary cause on which mental aberration, and all the consequences of that aberration, depend. Hence, if we admit the *effects* of a disease of the brain to continue after its primary cause shall have been fully removed, we at once break down all connexion between *cause* and *effect;* and, by so doing, we make an effect which, by its name, we acknowledge to be dependent to exist, while we suppose the cause on which it depends to be non-existent.

Can an effect continue in existence without a cause? This surely must be impossible. Can anything result from a cause which is admitted to be extinct? This must be as impossible as the other. Can anything which has in itself no independent existence derive a continuance of existence from itself? Neither can this be possible.

In admitting the FIRST of these cases, we must presume what we have denominated an effect to be an effect, and not an effect, at the same time, which is an evident contradiction. In admitting the SECOND case, we must presume that a cause can act after its non-existence, which is also a contradiction. And in admitting the THIRD case, we must ascribe independence to an effect which, from its name and nature, must be destitute of it, which is, in point of fact, denominating it to be independent, and not independent at the same time. Consequently, we conclude that as nothing in the FIRST CASE can be an effect without a cause; and in the SECOND, that no cause can act which is devoid of being; and that in the THIRD CASE nothing can derive from itself an independence which it does not possess—that no such instance can possibly exist. Besides, it is evident that no contact can exist between an effect which is in being, and a cause which is not; for if such contact can exist, then entity must depend on non-entity for the continuance of its existence, which is a self-evident absurdity.

Further, if the removal of the cause of any given effect produce no change in the state of that effect, it must be the removal of a cause which has no necessary connexion with its own effect; but since both the existence and removal of such a cause imply a contradiction, neither the existence nor the removal of a cause that produces no change in its own effect can possibly be admitted.

But if insanity be partly physical and partly spiritual in its character, then it must have two proximate causes—the one spiritual, the other physical, which is impossible.

*The supposition severs all connexion between cause and effect—i. e.,* it perpetuates existence without a cause, it makes entity to result from non-entity, and that which has only an independent existence is made to possess an independent one.

*Moreover, such a notion lays an interdict on recovery.*—How can the cure of mental aberration be ever effected if it has a proximate physical and a proximate spiritual cause? The means capable of effecting the one are directly antagonistic to the other; they are extremes "wide as the poles asunder," and if we could suppose either proximate cause the subject of removal, the insanity will be persistent; for it is the removal of a cause which has no necessary connexion with its own effect.

This contradictory supposition does not stop here; it involves considerations of the highest and most momentous importance in insanity —viz., the mode of its existence! A proximate physical and a proximate spiritual cause involves, as we have seen, the grossest absurdities, which another view of the subject will enable us to see more clearly. It is self-evident that a mere non-entity can never act, which the hypothesis in question supposes different. The existence of insanity must, therefore, be either *real* and *absolute*, or relative and dependent, or a mere privation; these being the only modes of possible existence of which we have any conception.

Whether "*mental aberration*" has a *real* or only a *relative* existence, or whether it is considered in no other light than that of a mere *privation*, the reasonings which have been adduced to prove that it must cease to exist when disease of the brain shall be cured, will equally apply, and clearly prove in either case, that as disease of the brain is its primary cause, the bounds of its duration must be limited to the cure of that morbid condition of the cerebral structure thus implicating the mental attributes.

That intellectual insanity is but *relative*, and therefore destitute of all positive existence, is a matter of absolute certainty. To maintain, therefore, the purely "spiritual character of insanity," I agree would be an "extreme opinion."

But we have before proved, that every form of disturbed mental harmony depends on disease of the brain, consequently insanity, whether *real* or *relative*, or whether only a mere *privation*, can have no further existence than the cause, modified or not, on which it depends.

If the existence of intellectual insanity be only relative, and consequently one with which the notion of positive existence can have no connexion, it will involve a contradiction to suppose that it can survive the cause which gave it birth, and on which it must be dependent for its mode of existence.

To presume that intellectual insanity could survive the cause which produced it, and on which it must continually depend, it will no longer be a relation, but a positive being. And to suppose anything can have a positive existence which is admitted to be but a mere relation, is to suppose that it is a relation, and not a relation at the same time.

But endeavouring to SHARE these "*extreme opinions*" respecting the character of insanity, virtually constitutes it a *positive existence;* for if insanity be neither altogether physical nor altogether spiritual in its character, then by a parity of reason it must be partly the one and partly the other, which is impossible.

As the cause of intellectual insanity in all its important bearings is to be referred to cerebral disease, it is evident that this disease must be removed in order to establish the sanity of the mind, and the possibility of this is placed beyond doubt, as the mental disturbance possesses only a relative existence.

Whatever results from a relation must, from the circumstances of that relation, necessarily be in a dependent state; for we can no more conceive that a mere relation can exist abstracted from that subject, from which it derives its existence, than we can conceive a shadow to exist when its only occasion is totally destroyed. When, therefore, the physical causes of intellectual insanity shall be cured, the normal character of the mind must be restored; and if these causes ceased to be causes, the normal character of the mental attributes would never be infringed upon!

The truth of the proposition, that insanity is altogether physical in its character, will receive additional corroboration if we consider it in the light of a *mere privation.* In short, a mere privation, in this view of the subject, is but a branch of relative existence, and is therefore connected with it. The same observation will apply in both cases, and the cure of mental aberration, whether considered either as a mere relation or as a privation of any particular mode of the mind's operations, must be the cure of this relation or of that privation; and consequently, that which cures my privation of mental sanity restores me again to absolute mental sanity, and banishes that privation in which my insanity of mind consisted.

If a privation of the normal manifestation of the mental attributes date its origin from any given cause, it is certain, whatever the nature of the cause may be, that it can only possess a dependent mode of existence, and that it can continue no longer in existence than it is supported by the cause on which it depends. And as the cure of the cause must cure all dependencies, the privation of mental insanity must result; and consequently where the absence or privation of mental sanity is not observable, sanity of mind must be in a state of actual existence.

It follows, therefore, that the cure of mental insanity must be a restoration unto mental sanity, and a restoration of the particular disease of the cerebral structure on which this mental condition depends.

That insanity is purely physical in its character, will be evident, if we view it in the light of a mere negation. The removal of a negation must be the production of positive existence; and it is only by the introduction of the latter, that the former can be effected. The removal of darkness must be the introduction of light; and we can no more conceive that a medium state can exist between them, in which neither light nor darkness makes its appearance, and naturally exists, than we can conceive how any given portion of space can be deprived of existence, or that matter can exist without figure or extension. As, therefore, there be no medium between a normal and abnormal state of any given structure, it follows that the removal of the one must be the introduction of the other, just as the removal of light must be the introduction of darkness as an inevitable consequence.

If, then, the negation of mental sanity is the identical cause which introduces mental insanity, so the removal of this negation of mental sanity must be the removal of mental insanity; and the removal of mental insanity, must be the identical cause which restores to mental sanity.

The removal of a negation must be the introduction of the reverse; without this, no removal of a negation can be supposed. If, then, mental insanity be a negation of mental sanity, and this negation be removed, if the removal of this negation be the identical cause which introduces the reverse, it follows that the removal of mental sanity is the removal of the absence of sanity, and is consequently the very cause through which mental sanity must be restored.

Now, since this privation of mental sanity, which exists to a lamentable extent, must be occasioned by some cause, it must necessarily be dependent; because it will involve a contradiction to suppose that a mere negation can exist in any other mode. If, therefore, the privation of mental insanity be dependent, and dependent on that cause which called it into existence, the cure of this cause must necessarily occasion the cure of this privation of mental sanity; and the instant in which it is effected, it must give place to that mental sanity which is the reverse. For, since in the consideration now before us, the reverse of mental sanity must be mental insanity, or the privation of mental sanity, so the cure of this mental insanity, or privation of mental sanity, must be the identical cause which restores mental sanity, it follows, therefore, that when those suffering from mental insanity shall be delivered from the captivity of the disease of their cerebral structure, they must exhibit every feature of mental sanity. The cure of intellectual insanity has been shown throughout to be dependent on the cure of the existing disease of the brain, in contradistinction to those that would constitute the mental condition (if possible) in part, spiritual in its character; and at the same time to illustrate the value of, and the necessity of, perseverance in the use of appropriate means of cure.

How necessary, then, is it for us to support, by every argument which reason can furnish, those principles and distinctions which Nature (or rather, which God) has presented to our notice; for Seneca says, "Whatever *Nature* does, *God* does."

But the supposition that insanity is partly spiritual in its character, involves other weighty and important considerations. If the mind be subject to derangement, it could neither be immaterial nor immortal; and if it could be deranged independently of the brain, by what method of treatment would it ever be cured? No reasoning, or appeals to the understanding, would be of service; and besides, in cases where it has been attempted, injurious results have been the consequence. The phrase, therefore, "*derangement of mind*," conveys an erroneous idea; for such derangement being only a characteristic of a particular disease of the brain, cannot be referred exclusively to the mind.

To this view of the subject, it is objected, that we "overlook the great power of psychical and moral influences," which is "*apt to give rise to the coarse, empirical, and dangerous practice of excessive bleedings, purgatives, vomitings*," &c. This is a gratuitous assumption. Besides, it is absurd to speak of "psychical and moral influences" as militating against the truth of the arguments which have been adduced in support of the physical character of insanity, when they have really nothing to do with the point at issue. These "influences" come not within the circuit of our considerations, unless they take the rank of exciting or proximate causes to that physical condition of the brain on which "mental aberration" supervenes.

An "*influence*," in a medical point of view, is only another word for a *cause;* and it is only by inducing some effect, that we are able to recognise its character as an influence in any particular relation in which it is regarded.

Now, if "mental insanity" be not removed, or cease to exist when disease of the brain is cured, the mind must be detained in this insane state by some power, or it must not. If by some power or influence, it is evident that this power or influence must partake of the mental disturbance, because that which has no connexion with the mental insanity can never detain the mind in a state of insanity. It therefore follows, that the instant we suppose the mind to be detained in an insane condition by any active power, we at once attribute the detaining power to the physical disease, which we have presumed removed, and suppose a connexion to subsist between that which is, and that which we admit to have been removed or cured. In short, it is to attribute the detaining power to disease of the brain, and not to attribute it at the same time, which is a palpable contradiction. On the contrary, if the minds of the mentally insane are detained in an insane state by no power, the argument is defeated, and operates in favour of a restoration—*i. e.*, by the aid of means calculated to remove the physical disease of the brain—from this mentally insane condition. For, since that which is divested of power can produce no effects, to suppose that the restoration of the mentally insane can be prevented through a *mere negation*, is to suppose the mind to be detained in an insane state by a nonentity—*i. e.*, no disease.

We therefore conclude that mental *insanity* is purely physical in its character.

*Braunston, Northamptonshire.*

---

